# Novel *CARMIL2* loss-of-function variants are associated with pediatric inflammatory bowel disease

**DOI:** 10.1038/s41598-021-85399-9

**Published:** 2021-03-15

**Authors:** Luca Bosa, Vritika Batura, Davide Colavito, Karoline Fiedler, Paola Gaio, Conghui Guo, Qi Li, Antonio Marzollo, Claudia Mescoli, Ryusuke Nambu, Jie Pan, Giorgio Perilongo, Neil Warner, Shiqi Zhang, Daniel Kotlarz, Christoph Klein, Scott B. Snapper, Thomas D. Walters, Alberta Leon, Anne M. Griffiths, Mara Cananzi, Aleixo M. Muise

**Affiliations:** 1 Department of Woman’s and Child’s Health, University of Padova, 35128 Padua, Italy; 2 SickKids Inflammatory Bowel Disease Centre, The Hospital for Sick Children, 555 University Ave, Toronto, ON M5G 1X8 Canada; 3 Research & Innovation (R&I Genetics) Srl, C.so Stati Uniti 4, 35127 Padua, Italy; 4 Pediatric Hematology, Oncology and Stem Cell Transplant Division, Padova University Hospital, 35128 Padua, Italy; 5 Fondazione Città della Speranza, Istituto di Ricerca Pediatrica, 35127 Padua, Italy; 6 Department of Medicine, Padova University Hospital, 35128 Padua, Italy; 7 Division of Gastroenterology and Hepatology, Saitama Children’s Medical Center, 1-2 Shintoshin, Chuo-ku, Saitama, Saitama 330-8777 Japan; 8 Department of Pediatrics, Dr. von Hauner Children’s Hospital, University Hospital, LMU Munich, Munich, Germany; 9 Division of Gastroenterology, Hepatology and Nutrition, Boston Children’s Hospital, Harvard Medical School, Boston, MA USA; 10 Division of Gastroenterology, Brigham and Women’s Hospital, Boston, MA USA; 11 Department of Paediatrics, University of Toronto, The Hospital for Sick Children, Toronto, ON M5G1X8 Canada; 12 Cell Biology Program, Research Institute, The Hospital for Sick Children, Toronto, ON M5G0A4 Canada

**Keywords:** Genetics, Chronic inflammation

## Abstract

CARMIL2 is required for CD28-mediated co-stimulation of NF-κB signaling in T cells and its deficiency has been associated with primary immunodeficiency and, recently, very early onset inflammatory bowel disease (IBD). Here we describe the identification of novel biallelic *CARMIL2* variants in three patients presenting with pediatric-onset IBD and in one with autoimmune polyendocrine syndrome (APS). None manifested overt clinical signs of immunodeficiency before their diagnosis. The first patient presented with very early onset IBD. His brother was found homozygous for the same *CARMIL2* null variant and diagnosed with APS. Two other IBD patients were found homozygous for a nonsense and a missense *CARMIL2* variant, respectively, and they both experienced a complicated postoperative course marked by severe infections. Immunostaining of bowel biopsies showed reduced CARMIL2 expression in all the three patients with IBD. Western blot and immunofluorescence of transfected cells revealed an altered expression pattern of the missense variant. Our work expands the genotypic and phenotypic spectrum of CARMIL2 deficiency, which can present with either IBD or APS, aside from classic immunodeficiency manifestations. *CARMIL2* should be included in the diagnostic work-up of patients with suspected monogenic IBD.

## Introduction

The *CARMIL2* gene (Capping Protein Regulator And Myosin 1 Linker 2, also known as *RLTPR*) is located on chromosome 16 and encodes a cell membrane-cytoskeleton-associated protein expressed in many cell types, including bone marrow and lymphoid tissue, endocrine glands and the gastrointestinal tract^1^. CARMIL2 controls actin polymerization at the barbed end of actin filaments, thus regulating a variety of cell functions related to membrane-associated actin assembly and signaling (e.g. cell morphology, polarity, protrusion formation and migration)^2–7^. Independently of its actin-uncapping function, CARMIL2 is required for CD28-mediated co-stimulation of NF-κB signaling in T cells, which is important for naive T cells activation, proliferation, maturation into T memory cells, and differentiation into T helper (Th) and T regulatory cells (Treg). Acting as a scaffold, CARMIL2 couples CD28 to the CARD11/CARMA1 cytosolic adaptor, thereby activating the NF-κB signaling pathway^8,9^. Finally, CARMIL2 has a role in antigen-receptor signaling in B cells, leading to NF-κB activation after B cell receptor (BCR), but not CD40, ligation^10^.

*CARMIL2* variants have been implicated in human disease. CARMIL2 deficiency has been associated with an autosomal recessive primary immunodeficiency (Immunodeficiency 58 IMD58 [MIM: 618131]), characterized by recurrent and/or chronic bacterial, viral, and fungal infections, cutaneous manifestations including eczematous dermatitis, and disseminated Epstein–Barr virus-associated smooth muscle tumors^9–14^. Recently, biallelic loss-of-function (LoF) variants of *CARMIL2* have been linked to very early onset inflammatory bowel disease (IBD) or IBD-like inflammatory gastrointestinal disorder, with or without clinical manifestations of immunodeficiency^11,13,15–18^.

IBD is a spectrum of complex multifactorial immune disorders characterized by chronic intestinal inflammation. Conventional IBD exhibits a polygenic architecture, while Mendelian or monogenic forms of IBD are caused by rare variants with a large effect on gene function^19–24^. The relative contribution of genetic factors and the frequency of IBD-causing monogenic variants seem to be inversely related to the age of onset of IBD^22,25–28^. A recent study identified rare variants in genes linked to monogenic IBD in 7.8% of subjects in the subgroup of very early onset IBD (VEOIBD), which includes patients younger than 6 years at disease recognition, and in 2.3% of children diagnosed with IBD after 6 years of age^29^. To date, about 70 monogenic defects affecting intestinal immune-epithelial homeostasis have been associated with IBD^29^. Among them, defects in T- and B-cell function and in Tregs and immune regulation can lead to immunodeficiency, autoimmunity, and IBD-like immunopathology^22^.

Autoimmune polyendocrine syndromes (APS) are characterized by functional impairment of multiple endocrine glands due to loss of immune tolerance toward them in genetically susceptible hosts. Similarly to IBDs, most APSs result from polygenic predisposition, although they rarely present as part of a broader syndrome with an underlying monogenic etiology. Monogenic APSs are caused by mutations in genes involved in maintenance of central (e.g. *AIRE*) or peripheral (e.g. *FOXP3*, *CTLA4*, *LRBA*, *STAT1*, *STAT3*, *STAT5b*, *ITCH*, *BACH2*) immune tolerance, leading to aberrant Treg function or activation of self-reactive effector T cells^30–33^.

Hereby, we describe the identification and characterization of novel *CARMIL2* variants in three patients diagnosed with pediatric-onset IBD and in a child affected by APS, thus expanding the genotypic and phenotypic spectrum of CARMIL2 deficiency.

## Results

### Identification of patients with biallelic *CARMIL2* variant

**Figure 1:**
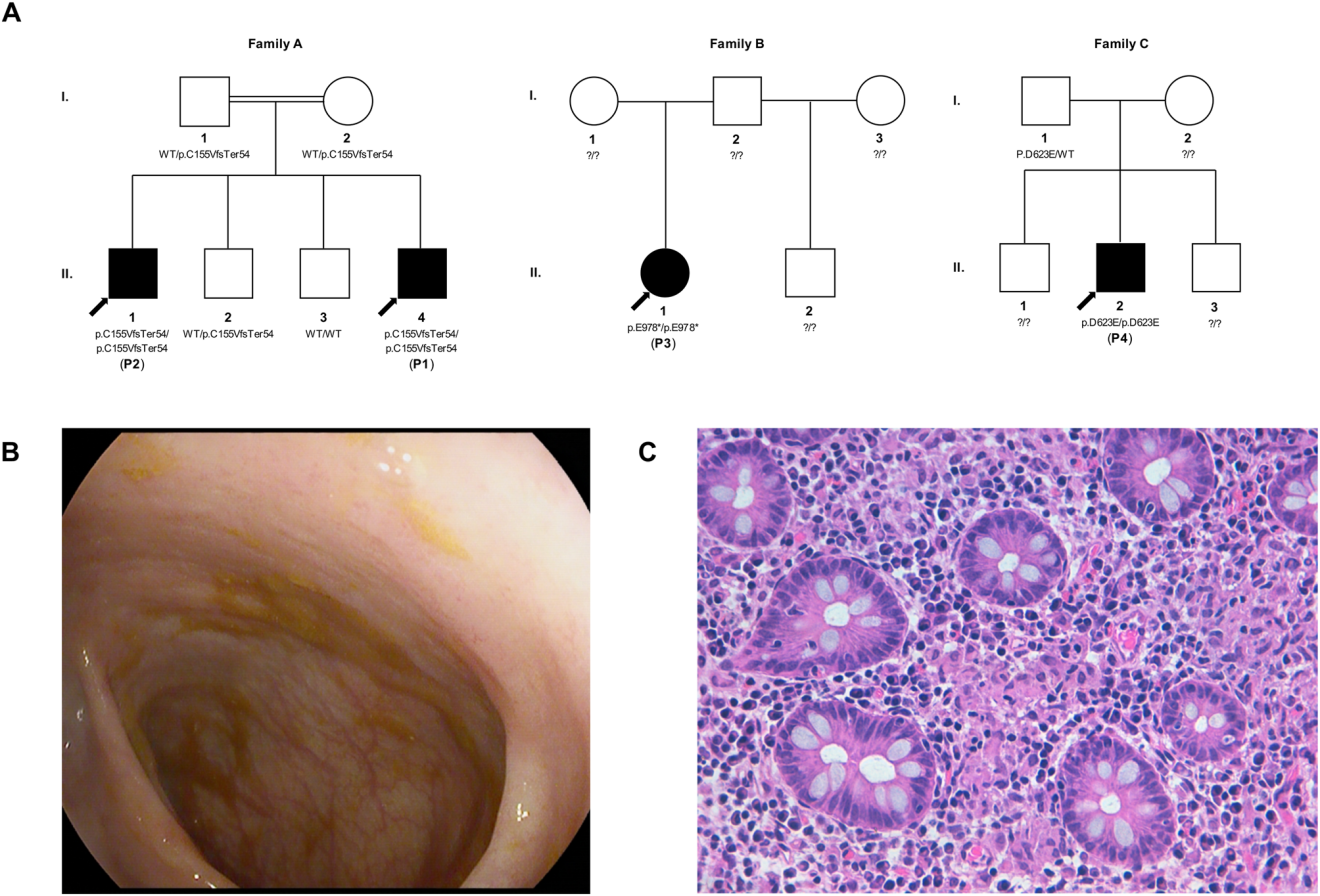
Novel biallelic CARMIL2 variants identified in four patients from three families. (**A**) Family pedigree and segregation analysis of the three kindreds. As for Family A, both parents and their second son, who had myoclonic epilepsy and attention deficit hyperactivity disorder, were heterozygous. (**B**) Colonoscopy image of Patient 1, showing only a mild patchy loss of vascular pattern throughout the colon. (**C**) Histopathologic findings in a colonic biopsy sample from Patient 1, showing epithelioid granulomas in the lamina propria (Hematoxylin–Eosin × 400).

In total, 4 patients from 3 unrelated families were identified with homozygous variants of *CARMIL2* (Fig. 1A). Patient 1 was identified through whole exome sequencing (WES), performed during the evaluation for suspected monogenic IBD at the University Hospital of Padova. Patient 2, the eldest brother of Patient 1, was recognized by family segregation analysis and then further sequenced by WES. Patient 3 and Patient 4 were identified by screening for biallelic *CARMIL2* variants from WES data in a cohort of 1005 pediatric IBD patients enrolled at SickKids, Toronto, as described in the “Methods”^29^.

### Clinical features of patients with biallelic *CARMIL2* variant

Patient 1 was born in Italy to consanguineous (first cousins) healthy Moroccan parents, fourth-born of four male sons. At 3.25 years he was diagnosed with colonic Crohn’s disease, phenotype A1aL2B1G1 according to the Paris classification (Fig. 1B,C, Table 1)^25^. Symptoms started when he was 2.6 years old. The patient was induced with prednisone, and after clinical remission he was maintained with azathioprine. Follow-up endoscopy performed after 18 months revealed histologic persistent mild total colonic inflammation in biopsies while in clinical remission. At the age of 5 years, the patient developed *Streptococcus pneumoniae* pneumonia complicated by sepsis, despite being vaccinated against pneumococcal disease. Past medical history included eczema in the first months of life. Anti-thyroglobulin antibodies and anti-thyroid peroxidase antibodies were significantly raised, while thyroid function tests and thyroid ultrasound were unremarkable.

**Table 1:** Demographic and clinical features of CARMIL2-deficient patients.

	Patient 1	Patient 2	Patient 3	Patient 4
**Demographics**				
Age at diagnosis	2 years	12 years	11 years	15 years
Gender	Male	Male	Female	Male
Consanguinity	Yes	Yes	No	Unknown
Parents origin (ethnicity/country)	Arab-Berber/Moroccan	Arab-Berber/Moroccan	Indigenous Canadian (mother), Israeli-Polish (father)	Unknown
**Gastrointestinal disease**				
Family history of IBD	None	None	None	None
IBD (Paris classification of IBD)^25^	CD (A1aL2B1G1)	None	UC (A1bE4S1)	UC (A1bE4S1)
Symptoms at onset	Chronic diarrhea, ± blood and mucus, ± abdominal pain, failure to thrive		Abdominal pain, bloody diarrhea, weight loss	Abdominal pain, bloody diarrhea, fever, weight loss
EGD/IC	Normal/mild patchy loss of vascular pattern		Gastric erythema and superficial erosion of duodenal cap/Pancolitis with normal terminal ileum	Hyperemic esophagus, small ulcers and inflammation of stomach/Severe pancolitis with backwash ileitis
Pathology (upper/lower gastrointestinal tract)	Altered villous profile and atrophy, mild lymphomonocytic inflammation and epithelioid microgranulomas in duodenal bulb/Crypt distortion, mild lymphomonocytic inflammation, Paneth cell metaplasia and epithelioid granulomas of the colon		Mild increase of duodenal lamina propria mononuclear cells, moderate antritis with focal acute activity, moderate chronic body gastritis/Mild-moderately active IBD of entire colon, focal superficial acute terminal ileitis	Normal duodenum, mild chronic non-atrophic gastritis, mild esophagitis/ Chronic colitis with mild to moderate acute activity throughout all biopsies
Surgery	None		Colectomy, ileostomy, J pouch	Colectomy, ileostomy, J pouch
**Other clinical features**				
Infections	*Streptococcus pneumoniae* pneumonia complicated by sepsis	*Tinea corporis*, prolonged upper respiratory tract infections	Intestinal leak with intraabdominal sepsis, pouchitis, pneumonia	Hepatic abscesses, *C. difficile* infection, abdominal abscess with enterocutaneous fistula
Endocrine disorders	None	Addison’s disease, Hashimoto thyroiditis	None	None
Skin	Eczema (first months)	Eczema	None	None
Others	Neuropsychiatric disorder (developmental delay and behavioral disorders)	Depressive disorder	Reactive airway disease	Unknown
Autoantibodies	Anti-thyroid Abs; negative anti-Harmonin antibodies	Anti-thyroid Abs, LAC	ANCA	ANCA

*EGD* esophagogastroduodenoscopy, *IBD* inflammatory bowel disease, *IC* ileocolonoscopy, *LAC* Lupus anticoagulant.

Patient 2 is the eldest brother of Patient 1 (Table 1). The boy was diagnosed with Addison’s disease at 12 years of age, and shortly after with Hashimoto's thyroiditis. No other endocrinopathies were detected. He suffered from difficult-to-treat eczema from a young age. He had no candidiasis, but at the age of 17 he was clinically diagnosed with extended *Tinea corporis* skin infection. Laboratory tests were significant for negative anti-adrenal antibodies and positive anti-thyroid autoantibodies. Plasma assay for very long-chain fatty acids was normal, thereby excluding peroxisomal disorders. Abdomen magnetic resonance imaging revealed bilateral adrenal hypoplasia. Sanger sequencing of the *AIRE* gene was non informative. Thus, Patient 2 was diagnosed with autoimmune polyendocrine syndrome type 2 (APS-2), or Schmidt syndrome, whose distinctive feature is Addison’s disease, associated with at least one among autoimmune thyroid disease and type 1A diabetes mellitus. There was neither history of significant or recurrent infectious episodes, nor evidence of bowel disease and fecal calprotectin was normal.

Immunological work-up of Patients 1 and 2 revealed a marked increase in the proportion of naïve T and B cells, with a reduction in memory T and B cells and marginal zone B cells. Patient 1 but not patient 2 had an increase in the number of double negative T cells (CD3 + CD4-CD8-TCRαβ+) (Supplementary Table S1). Both patients had negative anti-EBV IgG and IgM and EBV-DNA on peripheral blood. Both patients had low titers of specific antibody against tetanus and diphtheria toxoids.

Patient 3 is a female diagnosed with ulcerative colitis (UC), type pancolitis (A1bE4S1), at the age of 11 years old (Table 1). Remission was achieved after induction with prednisone, and maintained first with sulfasalazine, then with azathioprine. After relapse, she failed infliximab and at the age of 17 years underwent colectomy with loop ileostomy and J pouch. Her post-operative course was complicated by intestinal leakage causing intraabdominal sepsis, stricture at pouch anastomosis and pouchitis. Past medical history included reactive airway disease and an episode of pneumonia. Before IBD onset she had no other significant history of infections or immune-related diseases. She had no familial history of IBD.

Patient 4 is a male diagnosed with UC, type pancolitis (A1bE4S1), at the age of 15 years (Table 1). The patient was induced with IV methylprednisolone, became steroid dependent first, then steroid refractory; azathioprine was thus introduced. He developed azathioprine-induced pancreatitis with pseudocysts, hepatic abscesses and *C. difficile* infection. Drain insertion was complicated by peri-sigmoid abscess, colonic-peripancreatic enterocutaneous fistula and toxic megacolon. At the age of 16 the patient underwent subtotal colectomy and ileostomy, then J pouch surgery. Past medical history was unremarkable. Particularly, he had no previous history of infections, or immune-related diseases. He had no familial history of IBD.

### Genetic analysis of patients with biallelic *CARMIL2* variant

**Figure 2:**
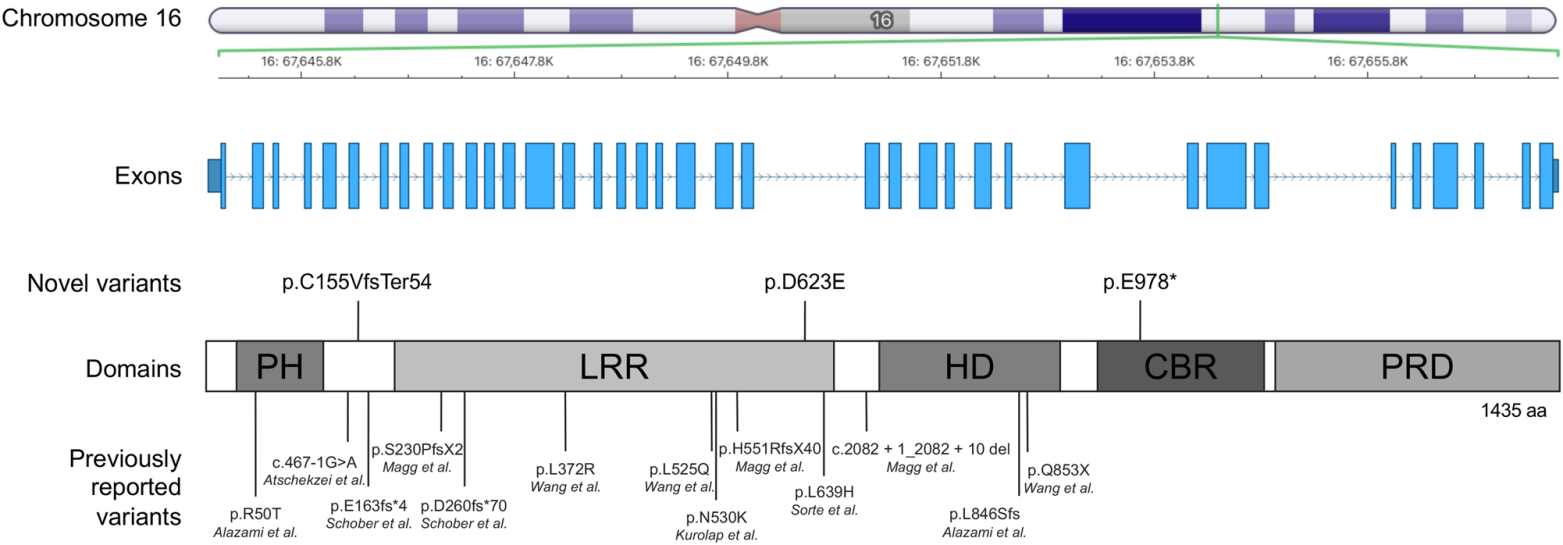
CARMIL2 gene and protein view with newly identified and previously published variants. Schematic representation of the intron–exon structure of the *CARMIL2* gene, which is located on Chromosome 16. The three novel variants reported in this study are labelled above the schematic illustration of the protein domains of CARMIL2, along with the previously described variants below. *PH* Pleckstrin-Homology domain, *LRR* Leucine-Rich Region, *HD* Helical Dimerization domain, *CBR* Capping Protein-Binding Region, *PRD* Proline-Rich Domain.

All four patients were found to have novel biallelic variants of *CARMIL2* (Fig. 2).

Patient 1 and 2 have a homozygous nonsense variant p.Cys155ValfsTer54 of *CARMIL2*, located on Exon 6 (Table 2). This frameshift mutation is caused by a single nucleotide (cytosine) deletion resulting in a nonsynonymous substitution of cysteine (C) with valine (V) and premature termination of translation after 54 codons (predicted number of amino acids: 207, while wild type CARMIL2 consists of 1435 amino acids). Variant p.C155VfsTer54 is not reported in literature and no data are available on allele frequency on reference databases (ExAC, gnomAD, 1000 Genomes database, EVS) (Table 2). Segregation analysis showed that the parents were heterozygous carriers of the variant.

**Table 2:** Genetic features of CARMIL2-deficient patients.

	Patient 1	Patient 2	Patient 3	Patient 4
Chromosome position^a^	Chr16:67,646,513		Chr16:67,653,066	Chr16:67,649,569
cDNA change (GenBank: NM_001013838.3)	c.462delC		c.2932G > T	c.1869C > A
Amino acid change^b^ (GenPept: NP_001013860.1)	p.Cys155ValfsTer54		p.Glu978*	p.Asp623Glu
Exon number	6		29	21
Predicted domain	None		PRD	LRR
**In silico evaluation**				
CADD	Unknown		41	26.4
SIFT Pred	Unknown		Unknown	Damaging
Polyphen2 Pred	Unknown		Unknown	Probably damaging
LRT	Unknown		Neutral	Deleterious
Mutation Assessor	Unknown		Unknown	Medium (2.645)
Mutation Taster	Disease causing		Disease causing	Disease causing
FATHMM	Unknown		Unknown	Tolerated
PROVEAN	Unknown		Unknown	Damaging
**Population databases**				
Maximum AAF^c^	0		0	0.0007
ExAC allele frequency	None		None	0.0002
N of heterozygous in ExAC	None		None	26
N of homozygous in ExAC	None		None	None
gnomAD allele frequency	None		None	0.0002499
gnomAD allele count	None		None	38
N of homozygous in gnomAD	None		None	None
1000 Genomes	None		None	None
EVS	None		None	0.0003

*AAF* alternative allele frequency, *AR* autosomal recessive, *CADD* Combined Annotation Dependent Depletion, *EVS* Exome Variant Server, *ExAC* Exome Aggregation Consortium, *FATHMM* Functional Analysis through Hidden Markov Models, *gnomAD* Genome Aggregation Database, *LRR* Leucine-Rich Region, *LRT* likelihood ratio test, *PRD* Proline-Rich Domain, *PROVEAN* Protein Variation Effect Analyzer.

^a^According to human genome assembly GRCh38.

^b^According to NCBI reference sequence NP_001013860.1.

^c^Represents the maximum AAF of a variant in the databases taken into account.

Patient 3 has a homozygous nonsense *CARMIL2* variant p.Glu978*, located on exon 29 of *CARMIL2* (Table 2) resulting in stop-gain nucleotide substitution and premature termination codon. *CARMIL2* variant p.E978* is not reported in literature and is absent from population databases. Patient 3 was recruited as a “singleton”, so parental DNA was not available for variant segregation analysis.

Patient 4 has a homozygous missense variant p.Asp623Glu, located on exon 21 of *CARMIL2* (Table 2). It has not been reported in literature, and its frequency is extremely low (< 0.001) in population databases. Variant p.D623E is predicted to be deleterious by the majority of the algorithms examined. Moreover, the aspartic acid at position 623 is located in a leucine-rich repeat (LRR) domain of CARMIL2 and is part of an evolutionarily constrained region (Supplementary Table S2). The patient was recruited as a “duo”, and only the unaffected father’s DNA was available for allele segregation analysis. The father was a heterozygous carrier for the same variant.

Sanger sequencing confirmed the presence of the biallelic variant p.C155VfsTer54 in Patient 1 and 2 but failed for both the variants identified in Patient 3 (p.E978*) and Patient 4 (p.D623E), due to a guanine-cytosine-rich template (Supplementary Fig. S1). However, careful reexamination of raw WES reads showed high coverage support for homozygous mutant calls in both candidates. Furthermore, since LoF is the pathogenic mechanism of CARMIL2 deficiency and tissue immunostaining proved reduced protein expression in both patients, confirmation by sequencing was deemed unnecessary^34^.

### Immunostaining of bowel biopsies in biallelic *CARMIL2* variant carriers with inflammatory bowel disease

**Figure 3:**
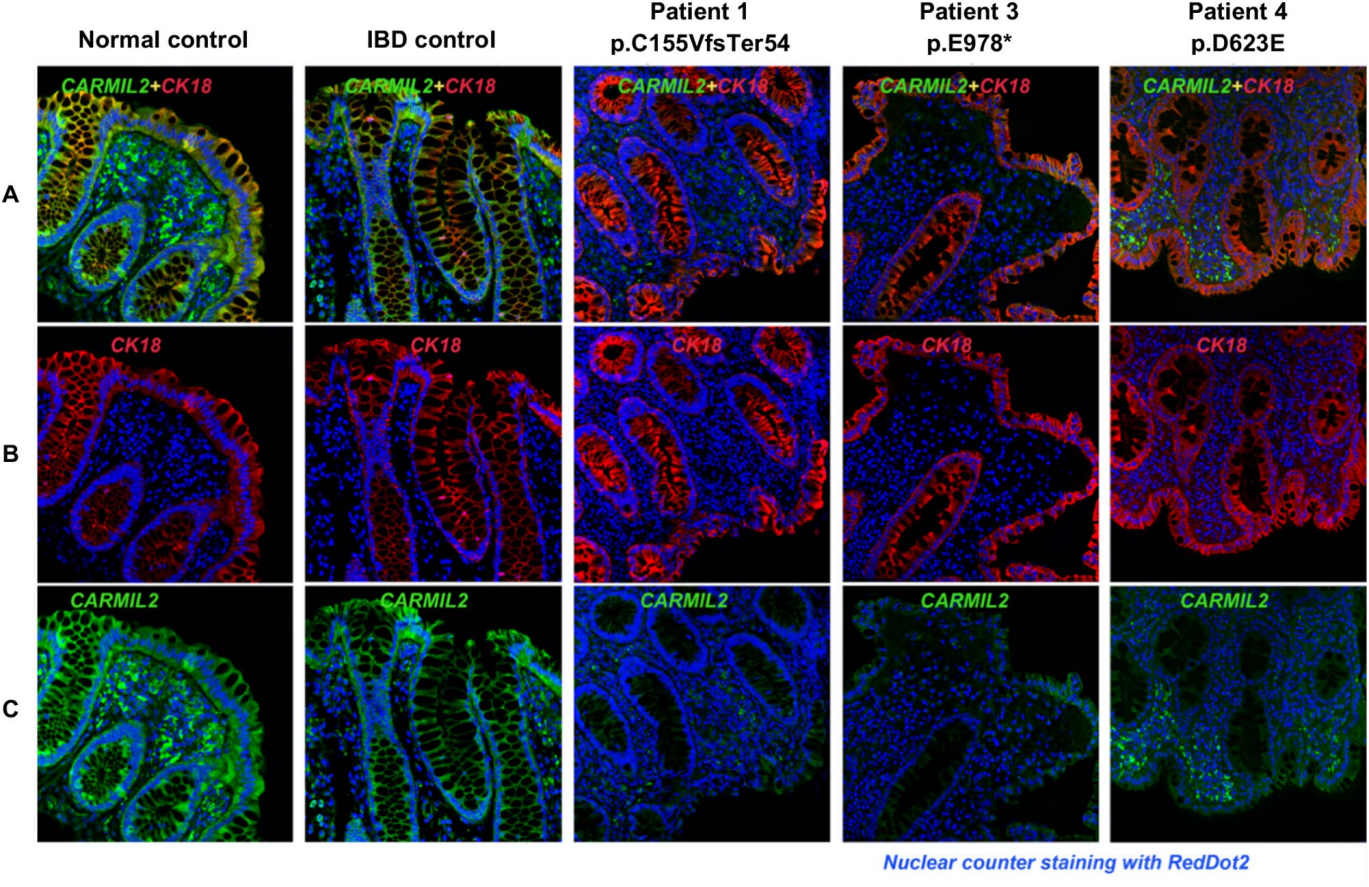
Dual immunofluorescence staining for CARMIL2 protein and CK18 on sigmoid FFPE sections of healthy control, IBD control, and three biallelic *CARMIL2* variant carriers diagnosed with IBD. DAPI counterstaining was used to visualize nuclei. (**A**) Composite image, where green staining indicates CARMIL2, red represents cytokeratin 18 (CK18), a marker for single layer epithelial cells, and blue marks nuclear DAPI stain. In Patient 1 and Patient 3, carriers of the two protein-truncating variants (respectively p.C155VfsTer54 and p.E978*), CARMIL2 signal was almost absent in sigmoid sections. In Patient 4, carrier of missense variant p.D623E, immunofluorescence staining was weaker than the controls particularly in the mucosal layer. (**B**) Single-label immunofluorescence for CK18 (red). (**C**) Single-label immunofluorescence for CARMIL2 (green).

Immunostaining on sigmoid formalin-fixed paraffin-embedded (FFPE) sections was performed to assess the expression levels of CARMIL2 in the bowel of the three patients affected by IBD (Patient 1, 3 and 4; Fig. 3). In both normal and IBD control sigmoid sections CARMIL2 was strongly expressed in the enterocytes of mucosal layer and stromal cells. In line with The Human Protein Atlas, CARMIL2 was mainly localized in cell cytoplasm, but some nuclear expression was also observed in blood cells^1^. In the two patients with homozygous protein-truncating variants (p.C155VfsTer54 and p.E978*), CARMIL2 protein expression was nearly absent or remarkably down-regulated in both glandular layer and stromal area. In Patient 4, carrying the homozygous missense variant p.D623E, CARMIL2 expression in the mucosal layer was significantly weaker in comparison to controls, while infiltrated blood cells located in the stromal area showed focally positive staining. This range of staining intensity in different cell types is consistent with previously published immunohistochemistry images, showing stronger staining in lymphocytes than in intestinal epithelium^18^. Cytokeratin 18 staining pattern in all samples was as expected based on previous reports^35^, indicating a preserved tissue architecture.

### Functional validation of *CARMIL2* variants

**Figure 4:**
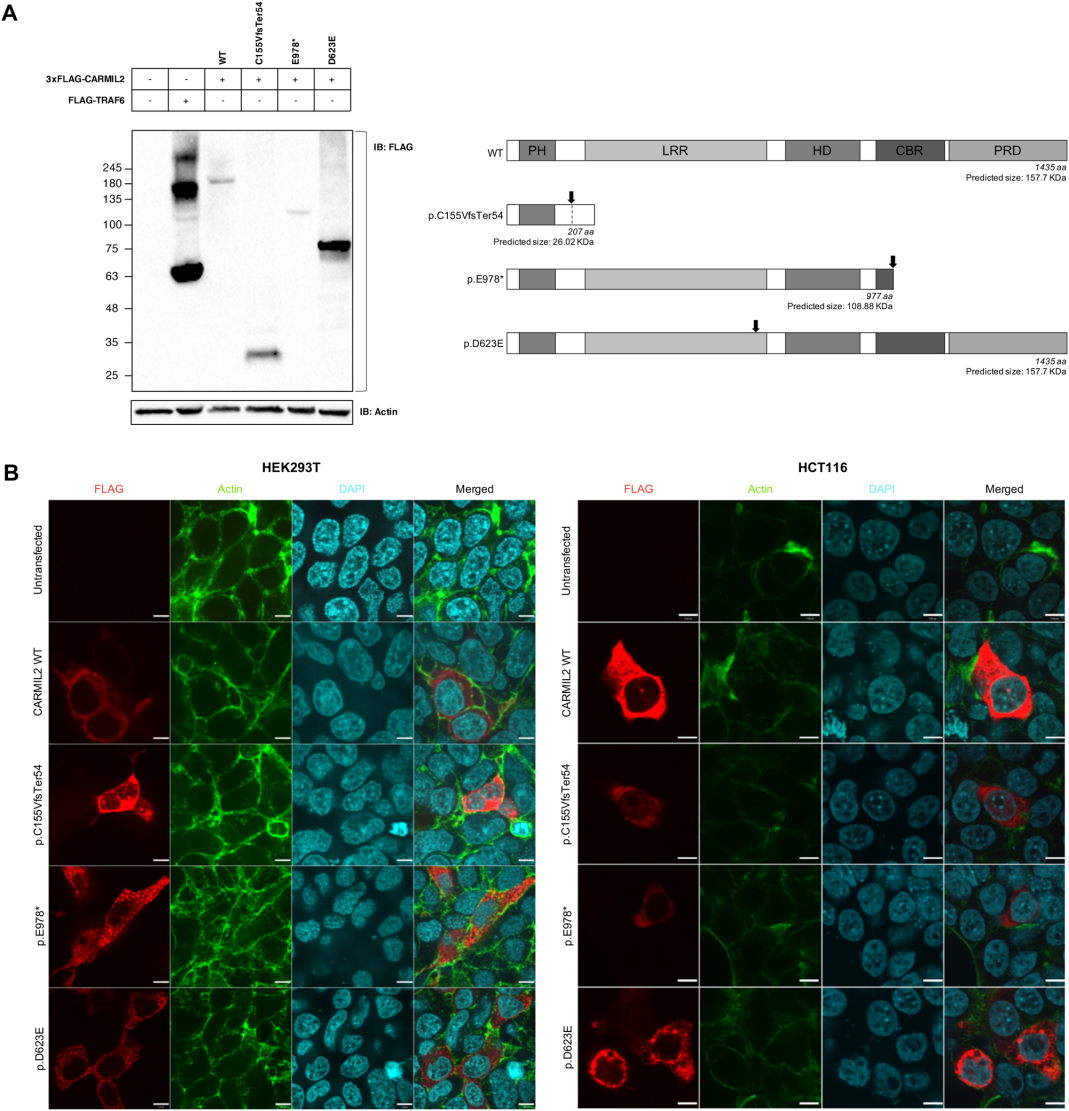
Functional validation of novel *CARMIL2* variants. (**A**) Western blot analysis of CARMIL2 expression. Anti-FLAG antibody was used for immunoblotting (left panel). FLAG-TRAF6 served as positive control, β-actin as loading control. The expected molecular weight of CARMIL2 WT and variants (considering the 3xFLAG tag) is shown for comparison (right panel). The black arrow points to the position of the mutation inside the protein. The observed band (~ 180 kDa) of WT CARMIL2 does not correspond to the predicted protein size (~ 155 kDa); this is consistent with what previously reported and with The Human Protein Atlas (available from v19.3.proteinatlas.org)^1,12^. Full-length blots are presented in Supplementary Fig. S2. (**B**) Immunofluorescence staining of HEK293T cells (left panel) and HCT116 cells (right panel) transfected with wild type CARMIL2 or the indicated variants. The first three columns represent the immunofluorescence images of FLAG tagged CARMIL2 (red), actin (green) and nuclei (light blue). The fourth column is a composite image. In HEK293T cells (left panel) CARMIL2 WT and variant p.C155VfsTer54 display a diffuse cytoplasmic expression pattern, while variants p.E978* and p.D623E exhibit a more granular pattern of expression. In HCT116 cells (right panel) expression pattern of all CARMIL2 isoforms is cytoplasmic, although variant p.D623E signal appeared as puncta structures. Scale bar: 7 µm.

Next, we investigated the impact of the novel *CARMIL2* variants on protein expression and cellular localization using cellular model systems. Western blotting was used to analyze the expression of 3xFLAG tagged CARMIL2 variants in HEK293Tcells (Fig. 4). Immunoblotting with anti-FLAG antibody (Fig. 4A) detected bands corresponding to the predicted molecular weight (MW) both for CARMIL2 wild type (WT) and for the protein-truncating variants, p.C155VfsTer54 and p.E978*. The MW of p.D623E variant was ~ 80 kDa, much lower than the WT, the only difference being the substitution of a single amino acid residue from aspartic acid to glutamic acid. This strongly suggested proteolysis of the p.D623E variant to a lower MW species.

Immunofluorescence staining of transfected cells was performed in both HEK293T cells and HCT116 cells (Fig. 4B). Immunofluorescence imaging showed that the expression pattern of CARMIL2 WT was diffuse across the cytoplasm in both cell strains, in line with the existing knowledge of the protein, as reported in The Human Protein Atlas^1^. Similarly, CARMIL2 variant p.C155VfsTer54 exhibited cytoplasmic expression, albeit less homogeneous. Conversely, CARMIL2 variants p.E978* and p.D623E signal appeared as puncta structures throughout the cytoplasm of HEK293T cells. For variant p.D623E the puncta staining was consistent in HCT116 cells. Therefore, this missense variant appeared to form aggregates when expressed in different cell lines. Overall, different lines of evidence suggest that variant p.D623E is susceptible to mislocalization inside the cell and proteolysis, thus behaving as a null or non-functional allele.

## Discussion

Our study confirms that CARMIL2 deficiency can manifest only with isolated IBD. Unlike more recent studies^17,18^ reporting a very early IBD onset (6 out of 6 patients), two of our patients shared a later age of onset of IBD symptoms, namely at 11 and 15 years of age. Although the majority of studies on monogenic IBD have focused on the very early onset population, running the risk of selection bias, recent findings have pointed out that a genetic disorder should be considered in all patients with pediatric onset IBD^29^. Resistance to conventional lines of treatment and complicated disease course, similar to what was reported in Patients 3 and 4, should always prompt the execution of WES, in line with existing recommendations^22^. None of our patients presented overt clinical signs of immunodeficiency before the diagnosis of IBD. Patient 1 developed bacterial sepsis after the initiation of immunosuppression (i.e., azathioprine). Notably, one patient reported in the study of Magg et al*.* died due to septic complications at the age of 4 years while on treatment with azathioprine^17^. Patient 3 and 4 experienced severe infectious complications after surgery. The need for a surgical treatment appears to be a common feature of pediatric IBD associated to CARMIL2-deficiency, since it occurred in the majority of the patients reported so far (6 out of 9 patients, including our cases) and was frequently due to a failure of medical treatment (3 out of 6 patients, including our cases)^17,18^. A recent study found that progression to surgery can be itself an indicator of monogenic etiology among pediatric IBD patients^29^. There is no consensus with respect to surgical timing, indications, and strategies in monogenic IBDs, including CARMIL2 deficiency^36^. Our results suggest that CARMIL2-deficient IBD patients harbor a significant risk of life-threatening immunosuppression- and surgery-related infections. A treatment option for several genetic immunodeficiencies underlying IBD-like phenotypes is hematopoietic stem cell transplantation (HSCT)^22^. However, it could be inappropriate or even harmful if an epithelial barrier defect coexists^23^. Herein, we confirm that CARMIL2 protein is expressed in gastrointestinal epithelium, but it remains to be clarified if this is relevant to IBD pathogenesis in deficient patients. Overall, *CARMIL2* should be included in the diagnostic work-up of patients with suspected monogenic IBD regardless of the age at disease onset and of the presence of overt clinical signs of immune deficiency. In fact, the identification of CARMIL2 deficiency has the potential to influence treatment choice and might improve disease prognosis, especially by means of prevention and prompt recognition of infectious complications.

Alongside phenotypic differences, CARMIL2 deficiency is characterized by marked genotypic heterogeneity. The known variants associated with CARMIL2 deficiency have different impact on the protein (e.g. nonsense, frameshift, missense, etc.) and they are spread along the gene (Fig. 2). The only consistency seems to be the lack of mutations in the C-terminus portion of the gene. One could speculate that mutations occurring in the C-terminus, close to end of the coding sequence, may preserve some level of protein function. Therefore, failure to produce the expected phenotype would lead to missed diagnosis. The lack of obvious genotypic-phenotypic correlation, as well as the inter- and intrafamilial clinical heterogeneity, even among carriers of the same *CARMIL2* variant, seems to point towards a contribution of additional environmental, genetic, or epigenetic-modifying factors in determining the clinical manifestations of CARMIL2 deficiency.

Bioinformatic tools can predict the damaging effects of mutations, but might overestimate them, hence the importance of experimental validation. Different lines of evidence from the experiments we performed showed that p.D623E variant behaves as a non-functional allele. Particularly, immunofluorescence of transfected cells resulted in a granular pattern. Endoplasmic-reticulum-associated protein degradation (ERAD) of p.D623E protein variant might explain this finding. In fact, the residue 623 of CARMIL2 belongs to an LRR domain, a structural motif with a horseshoe shape, with an interior parallel beta sheet, an exterior array of helices, and an hydrophobic core containing many leucine residues^37^. An amino acid substitution in this region may lead to the exposure of normally hidden hydrophobic patches, usually buried inside the protein to keep the lowest energy state. Exposed patches might lead to protein aggregation, or they could be recognized as a substrate by ERAD, a cellular pathway that targets misfolded or mutated proteins, which are retained inside the endoplasmic reticulum (ER) and targeted for ubiquitination and subsequent proteolytic degradation^38,39^. ER retention and impaired trafficking could explain the staining pattern observed in transfected cells, the proteolysis the lower than predicted MW. Protein overexpression might make the process more obvious by engulfing the cell. Another, non-exclusive possibility is that substitution from aspartic acid to glutamic acid leads the protein to be recognized by a glutamic-acid-specific protease.

This is the first report showing the association between CARMIL2 deficiency and autoimmune endocrinopathies. A monogenic etiology has been proved in a subset of patients with APS-2 and several lines of evidence support *CARMIL2* as a candidate gene^30–33,40,41^. First, the significance of rare LoF variants of *CARMIL2* in human disease has already been established, consistently with a recessive disease model^10^. This is confirmed by the modest LOUEF (loss-of-function observed/expected upper bound fraction) score (0.552 for *CARMIL2*, indicating a degree of intolerance to LoF variants) shown using gnomAD (Genome Aggregation Database) data^42^. Secondly, biallelic *CARMIL2* LoF variants fully segregate with an immune dysregulation disorder in multiple independent families with autoantibodies and absence of antigen-specific antibodies to bacterial vaccines^10,14^. This is consistent with the known B cell phenotype of human CARMIL2 deficiency. Additionally, skin manifestations have been described in the majority of CARMIL2-deficient patients, and eczema is part of other monogenic APSs^9–14,17,18,30^. Thirdly, the function of CARMIL2 is consistent with the known pathogenesis of APS, being expressed in immune cells and endocrine glands and being essential for development of regulatory T cells and for Th17 differentiation, similarly to other genes mutated in monogenic APS^1,8,9,30–33^. Moreover, *CARMIL2* interactome include *DOCK8*, that is mutated in patients presenting with autoimmune disorders, eczema and compromised Treg function^9,43^. Notably, CARMIL2-deficient mice and humans did not develop any obvious organ-specific autoimmune disorder, despite a reduction in Tregs^8,9^. This might depend on the coincident defect in effector T cells^9^. Furthermore, CARMIL2-deficient mice did not display any intrinsic B cell defect^8–10^. Therefore, knockout mice might not be a suitable model for studying human CARMIL2 deficiency. Remarkably, enteropathy and/or IBD-like intestinal inflammation, autoimmune endocrinopathy and skin manifestations are common in IPEX and other syndromes presenting with IPEX-like features, caused by monogenetic defects affecting Treg function^22,30,44^. Hence, CARMIL2 deficiency should be added to the increasing group of IPEX-like syndromes, as already proposed^18^.

Our study had some limitations, including that overexpression experiments might have been unreliable for protein-truncating variants p.C155VfsTer54 and p.E978*. In fact, it can be assumed that in vivo these variants are targeted by nonsense-mediated mRNA decay^45^. For this reason, the more intense immunofluorescence signal of p.C155VfsTer54 compared to WT CARMIL2 in cultured cells probably does not reflect what happens in vivo, and could have been determined by higher transfection efficiency due to a shorter peptide.

In summary, the phenotypic spectrum of CARMIL2 deficiency is broader than previously known, ranging from severe immunodeficiency to IBD and organ-specific autoimmunity. *CARMIL2* should be part of the diagnostic evaluation of patients with suspected monogenic IBD, even in the absence of obvious signs of immunodeficiency. Genetic diagnosis may be vital in monogenic IBD, to guide specific treatment, prevent surgery or unnecessary therapies, anticipate complications, and help genetic counseling^22,23,46–48^. Moreover, CARMIL2 deficiency can present exclusively with APS. Further studies are needed to better define IBD immunopathogenesis in CARMIL2 deficiency and the potential therapeutic utility of HSCT.

## Methods

### Helsinki guidelines

All human experiments followed the Helsinki Guidelines. Informed consent was obtained from the participants parents and the study had local ethics board approval at The Hospital for Sick Children (SickKids), Toronto, Canada (Research Ethics Board: REB1000024905).

### Patients

Patient 1 and his brother, Patient 2, were recruited from the Pediatric Gastroenterology, Hepatology and Liver Transplantation Unit at the Pediatric Clinic of the University Hospital of Padova. Patient 3 and Patient 4 were discovered through screening of a large cohort of pediatric IBD patients recruited at SickKids as previously described^29^. Children (age < 18) undergoing evaluation for IBD were enrolled over a 13-year period (2003–2015). Diagnosis of IBD was made according to the recommendations of the Porto criteria^49^. Patients with known primary immunodeficiency, chromosomal abnormalities, syndromic disease or diagnosed with other forms of monogenic intestinal disease were excluded. After obtaining the caregivers’ consent, clinical data were recorded, and biological samples were processed and stored in a biobank. WES analyses of blood samples was thus carried out as detailed below.

For Patient 1 and Patient 2, the phenotype review was carried out at University Hospital of Padova using electronic medical record software Galileo (NoemaLife, Dedalus). For the SickKids cohort, following variant prioritization^50^, each patient deemed to have pathogenetic biallelic *CARMIL2* variants was reversed phenotyped, using clinical data from databases and electronic medical record systems. For all IBD patients phenotype was classified according to the pediatric modification of the Montreal classification, also known as Paris classification^25^.

### Whole exome sequencing

For Patient 1 and 2, WES and segregation analysis were performed. Genomic DNA was extracted and purified from whole blood. DNA was prepared for sequencing with the kit SureSelectXT All Exon V5 kit (Agilent). Exome sequencing was performed with next-generation sequencing (NGS) technology on the Illumina HiSeq 2500 platform, using paired-end 100 bp read. DNA sequences were mapped and analyzed using as reference genome assembly GRCh38 (Genome Reference Consortium Human Build 38).

For the SickKids cohort, banked genomic DNA isolated from whole blood collected by venipuncture using a Qiagen Puregene Blood Core Kit was processed for exome capture using the NimbleGen VCRome 2.1 design. Captured libraries were sequenced on the Illumina HiSeq 2500 platform using paired-end 75 bp reads at the Regeneron Genetics Center (RGC), Tarrytown, NY, USA. Exome sequencing coverage was 30 × or greater for > 85% of the bases targeted.

### Bioinformatic analysis

For Patient 1, a bioinformatic analysis of exons and close intronic portions of 79 genes associated with IBD was performed (Supplementary Table S3). Genetic variants were analyzed and compared with several population databases (Exome Aggregation Consortium [ExAC], Genome Aggregation Database [gnomAD], 1000 Genomes database, Exome Variant Server [EVS]) and genetic clinical databases (OMIM, ClinVar, HGMD, GWAS, PGKB, Cosmic). Every variant was also evaluated in silico for its possible effects on protein structure or function (using Polyphen2, SIFT, VAAST Variant Prioritizer [VVP], Combined Annotation Dependent Depletion [CADD], Mutation Assessor, Mutation Taster) and for evolutionary conservation (phyloP score).

For the SickKids cohort, WES data from 2307 participants (1005 index patients, and 1302 parents and siblings) were analyzed using the FORGE (Finding of Rare Disease Genes) pipeline. Raw sequencing reads were aligned to human reference genome (GRCh38/hg38) using BWA-mem (Burrows-Wheeler Aligner, ver. 0.7.12), followed by indel realignment using Genome Analysis Toolkit (GATK, ver. 3.5). Five variant callers (GATK HaplotypeCaller ver. 3.5, Vardict ver. 1.4.6, Varscan ver. 2.3.9, Samtools ver. 1.3, and Freebayes ver. 1.0.0) were run on the Binary Alignment Map (BAM) files of each family to produce family based Variant Call Format (VCF) files when at least 2 of the 5 agreed on a called variant. Inheritance modeling on family level VCF files was performed using the GEMINI tool to query for rare (Minor Allele Frequency [MAF] < 0.01) protein coding variants that fit autosomal recessive, compound heterozygous, de novo, autosomal dominant, and X-linked inheritance models filters. VarSeq software (Golden Helix) was used to import, filter, and do inheritance modeling on the variants from each trio. Common variants (MAF > 0.01), defined using publicly available variant databases (ExAC frequency database [ver. 0.3], 1000 Genomes database [phase 1], and the NHLBI Exome Sequencing Project V2 Exome Variant Frequencies), were filtered out. Variants were further classified according to whether they were deemed to be coding. Non-synonymous and unclassified variants were then scored using the database for non-synonymous functional predictions (dbNSFP 2.8), filtering out variants with CADD score < 10 or no other damaging score (Polyphen2, SIFT, LRT, Mutation Assessor, Mutation Taster, Functional Analysis through Hidden Markov Models [FATHMM], Protein Variation Effect Analyzer [PROVEAN]). Variants were also evaluated in silico for protein domains in which they were predicted to be located (using Uniprot database and Simple Modular Architecture Research Tool or SMART database) and for evolutionary conservation of corresponding amino acid (based on Aminode webtool, available at http://www.aminode.org)^51^. Interpretation of all detected *CARMIL2* variants was performed according to the American College of Medical Genetics guidelines^52^.

### Sanger sequencing

For Patient 1, Patient 2 and their family, primers were designed to select and amplify through polymerase chain reaction (PCR) the region containing the reported mutation within the genomic DNA. PCR products were then sequenced using cycle sequencing Big Dye Terminator ver. 3.1 (Applied Biosystems) and ABI 3100 Avant automated capillary electrophoresis sequencer (Applied Biosystems).

For candidate patients identified within the SickKids pediatric IBD cohort, sequencing was carried out at The Centre for Applied Genomics (TCAG, The Hospital for Sick Children, Toronto, Canada), that provides high-quality capillary-based fluorescent sequencing on dual ABI 3730XL instruments. Different sets of primers were used to troubleshoot sequencing reactions.

### Immunofluorescence histochemical staining of bowel biopsies

Bowel samples were fixed in neutral buffered formalin without methanol and embedded in paraffin using routine protocols (formalin-fixed paraffin-embedded, FFPE). Tissue samples, including normal control and IBD control, were retrieved from the Unit of Anatomical Pathology, Medicine Department, University Hospital of Padova and from the Division of Pathology, The Hospital for Sick Children, Toronto. Only FFPE with well-preserved tissue architecture were chosen, to avoid false negative staining. As negative control and disease control, a non-IBD patient sample and an unrelated IBD patient sample were used, respectively. Immunofluorescent histochemical staining was performed on sigmoid FFPE sections as previously described^53^. Briefly, paraffin-embedded sections were deparaffinized using xylene and rehydrated with different percentages of ethanol. Antigen retrieval was achieved with high-pressure cooking in EDTA-borax buffer made with 1 mM EDTA, 10 mM borax, 10 mM boric acid and 0.001% ProClin 300 (Supelco) at pH 8.5. To block non-specific staining, the slides were incubated for 1 h at room temperature (RT) in 4% bovine serum albumin (BSA) and 20% donkey serum in phosphate-buffered saline (PBS). Slides were incubated with primary antibodies, including anti-CARMIL2 antibody produced in rabbit (Sigma, HPA041402) and anti-cytokeratin 18 mouse monoclonal antibody (Abcam), overnight at 4 °C. On the following day, stained slides were washed 3 times for 5 min with PBS. Secondary antibodies, namely Rhodamine Red-X (RRX) AffiniPure F(ab')_2_ Fragment Donkey Anti-Rabbit IgG (Jackson ImmunoResearch Laboratories) and Fluorescein (FITC) AffiniPure F(ab')_2_ Fragment Donkey Anti-Mouse IgG (Jackson ImmunoResearch Laboratories), were incubated at RT in darkness for 2 h, then slides were washed 3 times for 10 min in darkness. RedDot2 Far-Red Nuclear Stain (Biotium) was used for nuclear counterstaining at a dilution of 1:200. Finally, sections were mounted overnight with Vectashield antifade mounting medium (Vector Laboratories). Immunostained slides were imaged using a Leica confocal laser scanning microscope (Leica, TCS-SP8) and LAS-AF software (Leica Microsystems). Image processing, including color resolution, color separation, and merging of fields, was done using Adobe Photoshop CS5 software (Adobe Systems Incorporated).

### Plasmids

Plasmid containing an insert with human *CARMIL2* sequence with 3 tandem FLAG epitopes (DYKDDDDK) on N-terminus was a gift from John Cooper (Washington University, St. Louis, Addgene plasmid #118740)^5^. Mutations of *CARMIL2* were generated using site-directed mutagenesis by ACGT Corp. (Toronto, Canada). Plasmid were expanded through transformation and harvest from *Escherichia coli* DH10B bacteria as per standard protocols and purified using EZ-10 Spin Column Plasmid DNA Miniprep Kit (Bio Basic, BS614) and PureLink HiPure Plasmid Maxiprep Kit (Invitrogen, Thermo FisherScientific, K210007). Positive control plasmid FLAG-TRAF6 was provided by Muise laboratory (SickKids).

### Cell culture and transfection

HEK293T cells and HCT116 cells were provided by Muise laboratory and maintained in DMEM (Wisent Inc.) containing 10% of heat-inactivated fetal bovine serum (FBS) and antibiotic–antimycotics at 37 °C in 5% CO_2_. HEK293T cells were chosen for the ease of transfection, while HCT116 cells were selected for being a colon cancer cell line, thus closer to intestinal cells. For western blot, HEK293T cells were grown in a Falcon 6-well plate (Corning) to reach 60–70% confluence and transiently transfected with 1 µg of construct DNA (3xFLAG-CARMIL2 WT or mutation, or FLAG-TRAF6 as positive control) per each well using PolyJet (SignaGen Laboratories) transfection reagent, according to the manufacturer’s instructions. Cells were collected for lysis and protein analysis 48 h after transfection. For immunofluorescence, HEK293T cells and HCT116 cells were transiently transfected for 48 h as described above using 0.5 µg of construct DNA (3xFLAG-CARMIL2 WT or mutation) per well.

### Western blotting

Cells were lysed for 20 min in RIPA buffer (Sigma) supplemented with 1 mM phenylmethylsulfonyl fluoride (PMSF), 500 uM sodium fluoride (NaF), 0.2 mM sodium orthovanadate (Na_3_VO_4_), 1:500 Protease Inhibitor Cocktail (Sigma-Aldrich, P2714) and 1% Phosphatase Inhibitor Cocktail 3 (Sigma-Aldrich, P0044). Each sample was sonicated with 5 pulses at 30% amplitude (Q125 Sonicator, Qsonica) and centrifuged 20 min at 4 °C (Eppendorf Centrifuge 5430 R, Eppendorf). Protein concentration in lysate was measured using Bradford assay and a BSA protein standard in water (Bio-Rad Protein Assay Dye Reagent Concentrate, Bio-Rad; Epoch Microplate Spectrophotometer, BioTek). Samples were resuspended in 1xSDS (sodium dodecyl sulfate) protein sample buffer (40% glycerol, 240 mM Tris/HCl, 8% SDS, 0.04% bromophenol blue, 5% beta-mercaptoethanol) and a volume corresponding to 40 ug of protein per sample was loaded onto a 4–20% gradient gel (Mini-PROTEAN TGX Gels, Bio-Rad). Gel electrophoresis was performed (Mini-PROTEAN Tetra Vertical Electrophoresis Cell and PowerPac HC High-Current Power Supply, Bio-Rad) in running buffer (25 mM Tris base, 192 mM glycine, 0.1% SDS, pH 8.3) for approximately 1 h at 200 V. Sample was then transferred to a nitrocellulose membrane (Amersham Protran, GE Healthcare) using a semi-dry blotting process (Trans-Blot Turbo Transfer System, Bio-Rad) in transfer buffer (25 mM Tris base, 192 mM glycine, 0.00375% SDS, 20% (v/v) methanol, pH 8.3) for 12 min at 25 V and RT. Membrane was blocked using 5% skim milk in PBST (PBS with Triton X-100 0.05%) for 1 h at RT. The immunoblot was then incubated with appropriate primary and secondary antibodies diluted in blocking buffer, overnight at 4 °C and for 1 h at RT, respectively. Primary antibodies included monoclonal anti-FLAG M2 antibody produced in mouse (Sigma-Aldrich, F3165), anti-CARMIL2 antibody produced in rabbit (Sigma, HPA041402) and monoclonal anti-β-Actin antibody produced in mouse (Sigma-Aldrich, A5441). Secondary antibodies included Peroxidase AffiniPure Goat Anti-Mouse IgG (H + L) (Jackson ImmunoResearch Laboratories, 115-035-146), Peroxidase AffiniPure Goat Anti-Mouse IgG (H + L) (Jackson ImmunoResearch Laboratories, 115-035-003) and Peroxidase IgG Fraction Monoclonal Mouse Anti-Rabbit IgG light chain specific (Jackson ImmunoResearch Laboratories, 211-032-171). After incubation with each antibody, membranes were washed 3 times with PBST for 5–10 min. Blots were imaged using chemiluminescent horseradish peroxidase detection reagent (Immobilon Forte Western HRP substrate, MilliporeSigma or Clarity Max Western ECL Substrate, Bio-Rad) and imaged through chemiluminescence detection (ChemiDoc MP Imaging System and Image Lab software, Bio-Rad). In order to re-examine the same protein sample with different antibodies, stripping buffer (Restore Western Blot Stripping Buffer, Thermo Scientific, 21059) was added for 10 min. Membrane was then washed in PBST for 3 times and re-blocked. In total, the experiment was repeated 3 times. An online tool (available at https://www.bioinformatics.org/sms/prot_mw.html) was used to calculate the predicted MW of each CARMIL2 variant, taking into account the size of the protein tag (3xFLAG).

### Immunofluorescence staining of transfected cells

For immunofluorescence, HEK293T and HCT116 cells were cultured as described above, seeded at low density on cover slips (Fisherbrand) coated with poly-d-lysine (Sigma) onto Falcon 24-well plates (Corning) and transfected 1 h after seeding. After 48 h, cells where fixed in methanol-free 4% paraformaldehyde (PFA) for 30 min, then washed three times with PBS. Cells were permeabilized with 0.1% Triton X-100 in PBS for 10 min and blocked with 10% goat serum in PBS for 30 min at RT. Cells were incubated with the primary antibody, monoclonal ANTI-FLAG M2 antibody produced in mouse (Sigma, F1804) diluted in 10% goat serum PBS (1:200) for 1 h at RT. After washing 3 times with PBS-Tween (0.05%), cells were incubated with Mouse IgG (H + L) Highly Cross-Adsorbed Secondary Antibody, Alexa Fluor 568 Conjugate (Invitrogen, Thermo Fisher Scientific, A-11031) diluted in 10% goat serum PBS (1:200) for 45 min at RT, then washed again 3 times. 4′,6-diamidino-2-phenylindole (DAPI) diluted in PBS (1:2500) was used for nuclear staining, while actin was assessed via phalloidin staining (ActinGreen 488 ReadyProbes reagent, according to the manufacturer’s instructions); they were incubated together 10 min at RT. Cover slips were then washed with PBS and mounted onto glass slides (Fisherbrand) with mounting medium (Dako Fluorescent Mounting Medium, Agilent, S3023). Slides were imaged using a quorum spinning disk confocal microscope (Olympus IX81) set at 63 × objective magnification (oil imaging medium). Images were analyzed and deconvolved using Volocity 6.3 software (Perkin Elmer). For each condition, at least 3 images were acquired.

## Supplementary Information

Supplementary Information.
