# Supplementary material for: Novel CARMIL2 loss-of-function variants are associated with pediatric inflammatory bowel disease
Source: Sci Rep. 2021 Mar 15;11:5945. doi: 10.1038/s41598-021-85399-9 (PMC7960730; doi:10.1038/s41598-021-85399-9)
Supplement: Supplementary file 1 — Supplementary Information. [file 41598_2021_85399_MOESM1_ESM.docx]

**Supplementary Information**

**Novel *CARMIL2* loss-of-function variants are associated with pediatric inflammatory bowel disease**

Luca Bosa^1^, Vritika Batura^2^, Davide Colavito^3^, Karoline Fiedler^2^, Paola Gaio^1^, Conghui Guo^2^, Qi Li^2^, Antonio Marzollo^4,5^, Claudia Mescoli^6^, Ryusuke Nambu^2,7^, Jie Pan^2^, Giorgio Perilongo^1^, Neil Warner^2^, Shiqi Zhang^2^, Daniel Kotlarz^8^, Christoph Klein^8^, Scott B Snapper^9^, Thomas D Walters^2,10^, Alberta Leon^3^, Anne M Griffiths^2,10^, Mara Cananzi^1,+^ & Aleixo M Muise^2,10,11,*,+^

^1^Department of Woman’s and Child’s Health, University of Padova, Padova, 35128, Italy

^2^SickKids Inflammatory Bowel Disease Centre, The Hospital for Sick Children, Toronto, Ontario, M5G1X8, Canada

^3^Research & Innovation (R&I Genetics) Srl, C.so Stati Uniti 4, Padova, 35127, Italy

^4^Pediatric Hematology, Oncology and Stem Cell Transplant Division, Padova University Hospital, Padova, 35128, Italy

^5^Fondazione Città della Speranza, Istituto di Ricerca Pediatrica, Padova, 35127, Italy

^6^Department of Medicine, Padova University Hospital, Padova, 35128, Italy

^7^Division of Gastroenterology and Hepatology, Saitama Children’s Medical Center, 1-2 Shintoshin, Chuo-ku, Saitama-city, Saitama, 330-8777, Japan

^8^Department of Pediatrics, Dr. von Hauner Children's Hospital, University Hospital, LMU Munich, Munich, Germany

^9^Division of Gastroenterology, Hepatology and Nutrition, Boston Children’s Hospital, Harvard Medical School; Division of Gastroenterology, Brigham and Women's Hospital, Boston, MA, USA

^10^Department of Paediatrics, University of Toronto, The Hospital for Sick Children, Toronto, Ontario, M5G1X8, Canada

^11^Cell Biology Program, Research Institute, The Hospital for Sick Children, Toronto, Ontario, M5G0A4, Canada

^+^contributed equally to this work

^*^ **Correspondence** and requests for materials should be addressed to A.M.: Aleixo Muise MD, PhD, The Hospital for Sick Children, 555 University Ave., Toronto, ON, Canada, M5G 1X8. Email: [aleixo.muise@utoronto.ca](mailto:aleixo.muise@utoronto.ca). Phone: 416-813-7735. Fax: 416-813-6531

**Supplementary Tables**

**Table S1 Immunological work-up of Patient 1 and 2**

| **Parameter (unit)** | **Patient 1** | **Patient 2** |
| --- | --- | --- |
| Age | 4 years | 17 years |
| IgG (g/L) | 6.69 (5.52-11.98) | 11.89 (6.2-14.2) |
| IgA (g/L) | 1.53 (0.54-1.99) | 2.44 (0.50-3.00) |
| IgM (g/L) | 0.78 (0.40-1.45) | 1.39 (0.50-1.70) |
| Lymphocyte number (x 10^9/L) | 7.07 | 3.24 |
| CD3+ (%) | 58 (60-78) | 82 (63-80) |
| CD4+CD3+ (%) | 38 (31-47) | 47 (33-52) |
| CD8+CD3+ (%) | 17 (16-27) | 27 (19-29) |
| CD19+ (%) | 32 (13-29) | 15 (12-21) |
| CD16+CD56+(%) | 9 (5-16) | 3 (4-16) |
| CD4+CD45RA+ (% of CD4+ Ly) | 92 (61.8-85.0) ↑↑ | 86 (40.9-65.7) ↑↑ |
| CD4+CD45RO+ (% of CD4+ Ly) | 8 (14.8-37.2) ↓ | 14 (25.1-52.1) ↓ |
| CD3+CD25+ (%) | 1 (0.9-4.7) | 2 (3.8-7.8) |
| CD3+CD4-CD8-TCRαβ+ (% of CD3+ Ly) | 3.7 (< 2.5) ↑ | 0.55 (< 2.5) |
| Naive B IgD+CD27- (% of CD19+ Ly) | 97 (59.7-88.4) ↑↑ | 92 (61.6-87.4) ↑↑ |
| Marginal zone B IgD+CD27+ (% of CD19+ Ly) | 0.86 (3.1-17.4) ↓ | 0.7 (2.6-13.4) |
| Memory B IgD-CD27+ (% of CD19+ Ly) | 1.7 (2.9-17.4) ↓ | 2.8 (4.0-21.2) ↓ |
| Transitional B cells IgM+ CD38+ (% of CD19+ Ly) | 6.4 (2-30) | 20 (0.7-24) |
| Plasmoblasts IgM- CD38++ (% of CD19+ Ly) | 0.34 (0.1-4) | 0.65 (0.7-6) |
| Dihydrorhodamine test | Normal | NA |

**Table S2 Multiple sequence alignments of CARMIL-family proteins from diverse organisms containing the highly conserved aspartic acid residue 623 (sequence numbering is for human CARMIL2)**

|  |  |  |  |  |  |  |  |  |  |  | **623** | | |  |  |  |  |  |  |  |  |  |
| --- | --- | --- | --- | --- | --- | --- | --- | --- | --- | --- | --- | --- | --- | --- | --- | --- | --- | --- | --- | --- | --- | --- |
| **CARMIL2** | *Homo sapiens* | A | L | D | I | S | G | N | A | M | G | **D** | A | G | A | K | L | L | A | K | A | L |
|  | *Chlorocebus sabaeus* | A | L | D | I | S | G | N | A | M | G | **D** | A | G | A | K | L | L | A | K | A | L |
|  | *Nomascus leucogenys* | A | L | D | I | S | G | N | A | M | G | **D** | A | G | A | K | L | L | A | K | A | L |
|  | *Papio anubis* | A | L | D | I | S | G | N | A | M | G | **D** | A | G | A | K | L | L | A | K | A | L |
|  | *Callithrix jacchus* | A | L | D | I | S | G | N | A | V | G | **D** | A | G | A | K | L | L | A | K | A | L |
|  | *Otolemur garnettii* | A | L | D | I | S | G | N | G | M | G | **D** | V | G | A | K | M | L | A | K | A | L |
|  | *Tursiops truncatus* | A | L | D | I | S | G | N | A | M | G | **D** | T | G | A | K | M | L | A | K | A | L |
|  | *Equus caballus* | A | L | D | I | S | G | N | A | M | G | **D** | T | G | A | K | M | L | A | K | A | L |
|  | *Bos taurus* | A | L | D | I | S | G | N | A | M | G | **D** | T | G | A | K | M | L | A | K | A | L |
|  | *Myotis lucifugus* | A | L | D | I | S | G | N | A | M | G | **D** | T | G | A | K | M | L | A | K | A | L |
|  | *Ailuropoda melanoleuca* | A | L | D | I | S | G | N | A | M | G | **D** | T | G | A | K | M | L | A | K | A | L |
|  | *Felis catus* | A | L | D | I | S | G | N | A | M | G | **D** | T | G | A | K | M | L | A | K | A | L |
|  | *Ochotona princeps* | A | L | D | I | S | G | N | A | M | G | **D** | A | G | A | K | M | L | A | K | A | L |
|  | *Cavia porcellus* | A | L | D | I | S | G | N | A | M | G | **D** | A | G | A | K | M | L | A | K | A | L |
|  | *Pteropus vampyrus* | A | L | D | I | S | G | N | A | M | G | **D** | A | G | A | K | M | L | A | K | A | L |
|  | *Ovis aries* | A | L | D | I | S | G | N | A | M | G | **D** | T | G | A | K | M | L | A | K | A | L |
|  | *Rattus norvegicus* | V | L | D | I | S | G | N | A | I | G | **D** | T | G | A | K | M | L | A | K | A | L |
|  | *Erinaceus europaeus* | A | L | D | I | S | G | N | A | M | G | **D** | A | G | A | K | M | L | A | K | A | L |
|  | *Loxodonta africana* | E | L | D | I | S | G | N | A | M | G | **D** | T | G | A | K | M | L | A | K | A | L |
|  | *Oryctolagus cuniculus* | A | L | D | I | S | G | N | A | M | G | **D** | A | G | A | K | M | L | A | K | A | L |
|  | *Ictidomys tridecemlineatus* | S | L | D | I | S | G | N | A | M | G | **D** | T | G | A | K | M | L | A | K | A | L |
|  | *Dipodomys ordii* | A | L | D | I | S | G | N | A | M | G | **D** | A | G | A | K | M | L | A | K | A | L |
|  | *Sarcophilus harrisii* | S | L | D | I | S | G | N | A | M | G | **D** | T | G | A | K | M | L | A | K | A | L |
|  | *Monodelphis domestica* | S | L | D | I | S | G | N | A | M | G | **D** | T | G | A | K | M | L | A | K | A | L |
|  | *Meleagris gallopavo* | T | L | D | I | S | G | N | A | M | G | **D** | T | G | A | K | M | L | A | K | A | L |
|  | *Gallus gallus* | T | L | D | I | S | G | N | A | M | G | **D** | T | G | A | K | M | L | A | K | A | L |
|  | *Anas platyrhynchos* | A | L | D | I | S | G | N | A | M | G | **D** | T | G | A | K | M | L | A | K | A | L |
|  | *Macropus eugenii* | S | L | D | I | S | G | N | A | M | G | **D** | T | G | A | K | M | L | A | K | A | L |
|  | *Danio rerio* | K | I | D | I | S | G | N | C | I | G | **D** | T | G | A | K | M | L | A | K | A | L |
|  | *Lepisosteus oculatus* | K | I | D | I | S | G | N | L | I | G | **D** | T | G | A | K | M | L | A | K | A | L |
|  | *Xiphophorus maculatus* | E | L | D | I | S | G | N | N | I | G | **D** | T | G | A | K | M | L | A | K | A | L |
|  | *Xenopus tropicalis* | K | I | D | I | S | G | N | G | M | G | **D** | S | G | A | K | H | L | A | K | A | L |
| **CARMIL1** | ***Homo sapiens*** | K | V | D | I | S | G | N | G | M | G | **D** | M | G | A | K | M | L | A | K | A | L |
|  | *Pan troglodytes* | K | V | D | I | S | G | N | G | M | G | **D** | M | G | A | K | M | L | A | K | A | L |
|  | *Nomascus leucogenys* | K | V | D | I | S | G | N | G | M | G | **D** | M | G | A | K | M | L | A | K | A | L |
|  | *Callithrix jacchus* | K | V | D | I | S | G | N | G | M | G | **D** | M | G | A | K | M | L | A | K | A | L |
|  | *Otolemur garnettii* | K | V | D | I | S | G | N | G | M | G | **D** | M | G | A | K | M | L | A | K | A | L |
|  | *Equus caballus* | K | V | D | I | S | G | N | G | M | G | **D** | M | G | A | K | M | L | A | K | A | L |
|  | *Cavia porcellus* | K | V | D | I | S | G | N | G | M | G | **D** | M | G | A | K | M | L | A | K | A | L |
|  | *Loxodonta africana* | K | V | D | I | S | G | N | G | M | G | **D** | M | G | A | K | M | L | A | K | A | L |
|  | *Papio anubis* | K | V | D | I | S | G | N | G | M | G | **D** | M | G | A | K | M | L | A | K | A | L |
|  | *Chlorocebus sabaeus* | K | V | D | I | S | G | N | G | M | G | **D** | M | G | A | K | M | L | A | K | A | L |
|  | *Canis familiaris* | K | V | D | I | S | G | N | G | M | G | **D** | M | G | A | K | M | L | A | K | A | L |
|  | *Monodelphis domestica* | K | V | D | I | S | G | N | G | M | G | **D** | M | G | A | K | M | L | A | K | A | L |
|  | *Ictidomys tridecemlineatus* | K | V | D | I | S | G | N | G | M | G | **D** | M | G | A | K | M | L | A | K | A | L |
|  | *Ailuropoda melanoleuca* | K | V | D | I | S | G | N | G | M | G | **D** | M | G | A | K | M | L | A | K | A | L |
|  | *Rattus norvegicus* | K | V | D | I | S | G | N | S | M | G | **D** | M | G | A | K | M | L | A | K | A | L |
|  | *Mus musculus* | K | V | D | I | S | G | N | G | M | G | **D** | M | G | A | K | M | L | A | K | A | L |
|  | *Felis catus* | K | V | D | I | S | G | N | G | M | G | **D** | M | G | A | K | M | L | A | K | A | L |
|  | *Ochotona princeps* | K | V | D | I | S | G | N | A | M | G | **D** | M | G | A | K | M | L | A | K | A | L |
|  | *Tursiops truncatus* | T | V | D | I | S | G | N | G | M | G | **D** | M | G | A | K | M | L | A | K | A | L |
|  | *Bos taurus* | K | V | D | I | S | G | N | G | M | G | **D** | M | G | A | K | M | L | A | K | A | L |
|  | *Ovis aries* | K | V | D | I | S | G | N | G | M | G | **D** | M | G | A | K | M | L | A | K | A | L |
|  | *Echinops telfairi* | K | V | D | I | S | G | N | G | M | G | **D** | M | G | A | K | M | L | A | K | A | L |
|  | *Microcebus murinus* | K | V | D | I | S | G | N | G | M | G | **D** | M | G | A | K | M | L | A | K | A | L |
|  | *Anolis carolinensis* | K | V | D | I | S | G | N | G | M | G | **D** | M | G | A | K | M | L | A | K | A | L |
|  | *Meleagris gallopavo* | K | V | D | I | S | G | N | A | M | G | **D** | M | G | A | K | M | L | A | K | A | L |
|  | *Ficedula albicollis* | K | V | D | I | S | G | N | A | M | G | **D** | M | G | A | K | M | L | A | K | A | L |
|  | *Gallus gallus* | K | V | D | I | S | G | N | A | M | G | **D** | M | G | A | K | M | L | A | K | A | L |
|  | *Pelodiscus sinensis* | K | V | D | I | S | G | N | G | M | G | **D** | M | G | A | K | M | L | A | K | A | L |
|  | *Xenopus tropicalis* | K | V | D | I | S | G | N | G | M | G | **D** | M | G | A | K | M | L | A | K | A | L |
|  | *Latimeria chalumnae* | K | V | D | I | S | G | N | G | M | G | **D** | M | G | A | K | M | L | A | K | A | L |
|  | *Dipodomys ordii* | K | V | D | I | S | G | N | G | M | G | **D** | M | G | A | K | M | L | A | K | A | L |
|  | *Choloepus hoffmanni* | K | V | D | I | S | G | N | G | M | G | **D** | M | G | A | K | M | L | A | K | A | L |
|  | *Erinaceus europaeus* | K | V | D | I | S | G | N | G | M | G | **D** | M | G | A | K | M | L | A | K | A | L |
|  | *Poecilia formosa* | K | L | D | I | S | G | N | S | M | G | **D** | M | G | A | K | I | L | A | K | A | L |
|  | *Xiphophorus maculatus* | K | L | D | I | S | G | N | S | M | G | **D** | M | G | A | K | I | L | A | K | A | L |
|  | *Macropus eugenii* | K | V | D | I | S | G | N | G | M | G | **D** | M | G | A | K | M | L | A | K | A | L |
| **CARMIL3** | *Homo sapiens* | K | V | D | L | S | G | N | G | M | E | **D** | I | G | A | K | M | L | S | K | A | L |
|  | *Pongo abelii* | K | V | D | L | S | G | N | G | M | E | **D** | I | G | A | K | M | L | S | K | A | L |
|  | *Papio anubis* | K | V | D | L | S | G | N | G | M | E | **D** | I | G | A | K | M | L | S | K | A | L |
|  | *Chlorocebus sabaeus* | K | V | D | L | S | G | N | G | M | E | **D** | I | G | A | K | M | L | S | K | A | L |
|  | *Callithrix jacchus* | K | V | D | L | S | G | N | G | M | E | **D** | I | G | A | K | M | L | S | K | A | L |
|  | *Ictidomys tridecemlineatus* | K | V | D | L | S | G | N | G | M | E | **D** | I | G | A | K | M | L | S | K | A | L |
|  | *Otolemur garnettii* | K | V | D | L | S | G | N | G | M | E | **D** | I | G | A | K | M | L | S | K | A | L |
|  | *Bos taurus* | K | V | D | L | S | G | N | G | M | E | **D** | I | G | A | K | M | L | S | K | A | L |
|  | *Sus scrofa* | K | V | D | L | S | G | N | G | M | E | **D** | I | G | A | K | M | L | S | K | A | L |
|  | *Canis familiaris* | K | V | D | L | S | G | N | G | M | E | **D** | I | G | A | K | M | L | S | K | A | L |
|  | *Mustela putorius furo* | K | V | D | L | S | G | N | G | M | E | **D** | I | G | A | K | M | L | S | K | A | L |
|  | *Felis catus* | K | V | D | L | S | G | N | G | M | E | **D** | I | G | A | K | M | L | S | K | A | L |
|  | *Rattus norvegicus* | K | V | D | L | S | G | N | G | M | E | **D** | I | G | A | K | M | L | S | K | A | L |
|  | *Mus musculus* | K | V | D | L | S | G | N | G | M | E | **D** | I | G | A | K | M | L | S | K | A | L |
|  | *Myotis lucifugus* | K | V | D | L | N | G | N | G | M | E | **D** | I | G | A | K | M | L | S | N | A | L |
|  | *Oryctolagus cuniculus* | K | V | D | L | S | G | N | G | M | E | **D** | I | G | A | K | M | L | S | K | A | L |
|  | *Tursiops truncatus* | K | V | D | L | S | G | N | G | M | E | **D** | I | G | A | K | M | L | S | K | A | L |
|  | *Loxodonta africana* | K | V | D | L | S | G | N | G | M | E | **D** | I | G | A | K | M | L | S | K | A | L |
|  | *Cavia porcellus* | K | V | D | L | S | G | N | G | M | E | **D** | I | G | A | K | M | L | S | K | A | L |
|  | *Ailuropoda melanoleuca* | K | V | D | L | S | G | N | G | M | E | **D** | I | G | A | K | M | L | S | K | A | L |
|  | *Ovis aries* | K | V | D | L | S | G | N | G | M | E | **D** | I | G | A | K | M | L | S | K | A | L |
|  | *Sarcophilus harrisii* | K | V | D | L | S | G | N | G | M | E | **D** | I | G | A | K | M | L | S | K | A | L |
|  | *Ochotona princeps* | K | V | D | L | S | G | N | G | M | E | **D** | I | G | A | K | M | L | S | K | A | L |
|  | *Microcebus murinus* | K | V | D | L | S | G | N | G | M | E | **D** | I | G | A | K | M | L | S | K | A | L |
|  | *Pteropus vampyrus* | K | V | D | L | S | G | N | G | M | E | **D** | I | G | A | K | M | L | S | K | A | L |
|  | *Dipodomys ordii* | K | V | D | L | S | G | N | G | M | E | **D** | I | G | A | K | M | L | S | K | A | L |
|  | *Echinops telfairi* | K | V | D | L | S | G | N | S | M | E | **D** | I | G | A | K | M | L | S | K | A | L |
|  | *Macropus eugenii* | K | V | D | L | S | G | N | G | M | E | **D** | I | G | A | K | M | L | S | K | A | L |
|  | *Erinaceus europaeus* | K | V | D | L | S | G | N | G | M | E | **D** | I | G | A | K | M | L | S | K | A | L |
| **Invertebrate CARMIL** | XP_021699954.1_LRRC16A_Mosquito | V | L | D | I | T | G | N | L | M | G | **D** | I | G | A | R | L | L | A | K | A | L |
|  | NP_001260788.1_Fruit_Fly | K | L | D | I | S | G | N | F | M | G | **D** | V | G | A | R | L | L | A | K | A | L |
|  | XP_006568383.2_LRRC16A_Bee | I | L | D | I | S | G | N | Q | I | G | **D** | P | G | A | R | L | L | A | K | A | L |

**Table S3 Exome-based genetic testing performed for Patient 1**

| **Biological Category** | **Genes** |
| --- | --- |
| Hyper- & Auto- inflammatory disorders | *MEFV, MVK, NLRC4, NLRP12, PLCG2, SH2D1A* |
| Immunoregulation disorders | *AIRE, FOXP3, HPS1, HPS4, HPS6, IL10, IL10RA, IL10RB, IL2RA, STAT1, STXBP2, XIAP* |
| Phagocyte defects | *CYBA, CYBB, G6PC3, ITGB2, NCF2, NCF4, SLC37A4* |
| T- and B-cell defects | *ADA, AICDA, BTK, CD3G, CD40LG, COG6, CTLA4, DCLRE1C, DKC1, DOCK8, ICOS, IL21, IL2RG, LCK, LIG4, LRBA, NFKBIA, PIK3R1, RAG1, RAG2, RTEL1, TNFRSF13B, WAS, ZAP70* |
| Apoptosis defects | *CASP8, MASP2, TRIM22* |
| Epithelial barrier defects | *ADAM17, COL7A1, EGFR, EPCAM, FERMT1, IKBKG, TGFBR1, TGFBR2,* |
| Others | *ABCB1, ANKZF1, ARPC1B, ATG16L1, CD19, DUOX2, GUCY2C, IFIH1, IL23R, IL6, IL7R, IRF5, IRGM, NFAT5, NOD2, NOX1, RLTPR, TTC37, TTC7A* |
|  |  |

**Supplementary Figures**

**A**

**B**


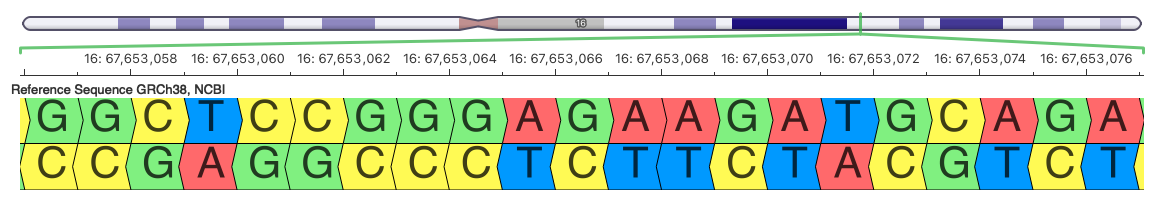


**Patient 3**

**c.2932G>T**


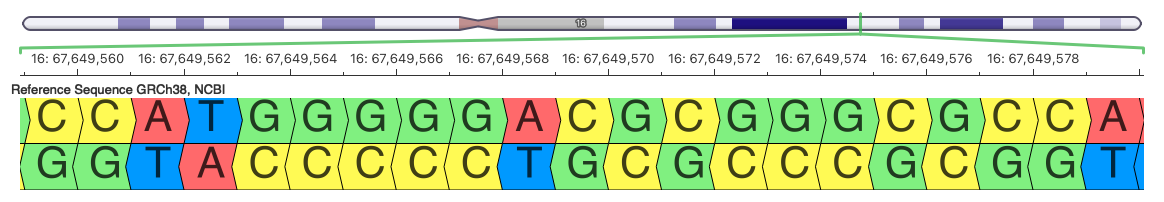


**Patient 4**

**c.1869C>A**

**Figure S1:** Reference nucleotide sequence of *CARMIL2* gene centered at position Chr16:67,653,066 (A) and Chr16:67,649,569 (B), where the variants of Patient 3 and Patient 4 are located, respectively


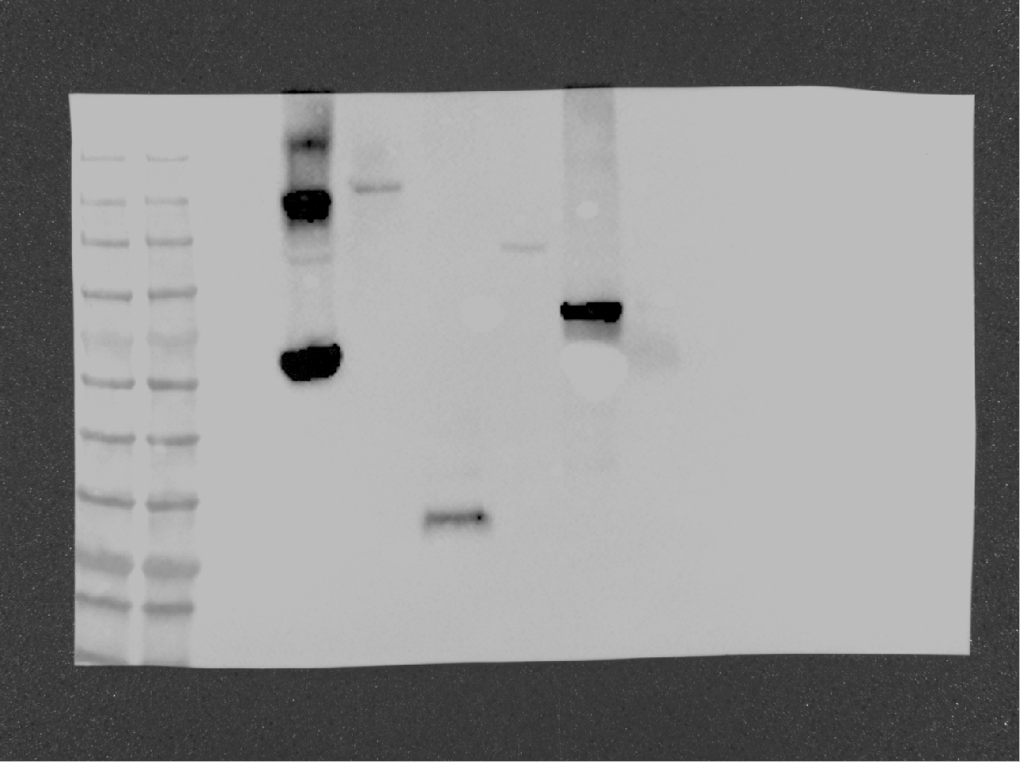


**IB: FLAG**


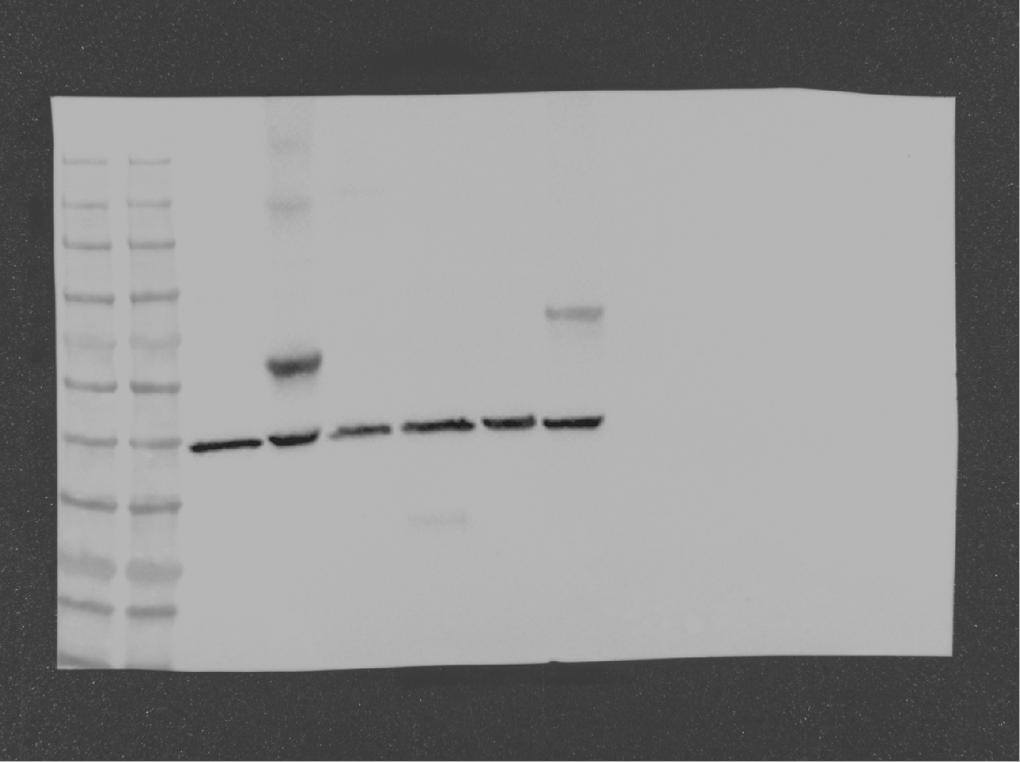


**IB: Actin**

**Figure S2:** Western blot analysis of CARMIL2 expression. Uncropped Western Blot images corresponding to Figure 4A. In order to re-examine the same membrane with different antibodies, stripping buffer was applied (hence, stronger bands from the first immunoblot could still be seen in the second one)
